# Dietary Flavonoid Hyperoside Induces Apoptosis of Activated Human LX-2 Hepatic Stellate Cell by Suppressing Canonical NF-*κ*B Signaling

**DOI:** 10.1155/2016/1068528

**Published:** 2016-03-27

**Authors:** Liwen Wang, Zhiwei Yue, Mengzheng Guo, Lianying Fang, Liang Bai, Xinyu Li, Yongqing Tao, Suying Wang, Qiang Liu, Dexian Zhi, Hui Zhao

**Affiliations:** ^1^Tianjin Key Laboratory of Food and Biotechnology, School of Biotechnology and Food Science, Tianjin University of Commerce, Tianjin 300134, China; ^2^Tianjin Key Laboratory of Radiation Medicine and Molecular Nuclear Medicine, Institute of Radiation Medicine, Chinese Academy of Medical Sciences and Peking Union Medical College, Tianjin 300192, China; ^3^Department of Hematology and Tangshan Key Laboratory, Translational Medical Center, North China University of Science and Technology, Tangshan, Hebei 063000, China

## Abstract

Hyperoside, an active compound found in plants of the genera* Hypericum *and* Crataegus*, is reported to exhibit antioxidant, anticancer, and anti-inflammatory activities. Induction of hepatic stellate cell (HSC) apoptosis is recognized as a promising strategy for attenuation of hepatic fibrosis. In this study, we investigated whether hyperoside treatment can exert antifibrotic effects in human LX-2 hepatic stellate cells. We found that hyperoside induced apoptosis in LX-2 cells and decreased levels of *α*-smooth muscle actin (*α*-SMA), type I collagen, and intracellular reactive oxygen species (ROS). Remarkably, hyperoside also inhibited the DNA-binding activity of the transcription factor NF-*κ*B and altered expression levels of NF-*κ*B-regulated genes related to apoptosis, including proapoptotic genes* Bcl-Xs*,* DR4*,* Fas*, and* FasL* and anti-apoptotic genes* A20*,* c-IAP1*,* Bcl-X*_*L*_, and* RIP1*. Our results suggest that hyperoside may have potential as a therapeutic agent for the treatment of liver fibrosis.

## 1. Introduction

Liver fibrosis is a major cause of morbidity and mortality worldwide due to chronic viral hepatitis and, more recently, from fatty liver disease associated with obesity [1]. Hepatic fibrosis is largely asymptomatic. However, progressive fibrosis resulting in cirrhosis can cause distortion of the liver parenchyma and vascular architecture [2], of which major clinical consequences are impaired liver function, an increased portal hypertension, and the development of hepatocellular carcinoma [3]. An epidemiological analysis has identified liver cirrhosis as a leading cause of disease-related death worldwide [4].

Liver fibrosis is a reversible, progressive pathological process characterized by excess accumulation of extracellular matrix (ECM) proteins. Hepatic stellate cells (HSCs) are quiescent, nonproliferative, vitamin-A storing cells; they are localized to the space of Disse and function as the principal storage sites of retinoids in normal liver. HSCs are the principal cell type involved in liver fibrogenesis, and the survival of activated HSCs is the hallmark feature of liver fibrosis. Upon activation, HSCs proliferate and undergo transdifferentiation from quiescent cells to activated myofibroblast-like cells secreting excess ECM proteins [5]. Antifibrotic drug research focuses on the inhibition of HSC activation or proliferation and the promotion of apoptosis in activated HSCs [6, 7]. Given the current lack of successful treatment options for liver fibrosis, new strategies for slowing this process are urgently needed.

In addition to the activation of fibroblasts and immune cells, fibrosis-inducing events also cause release of profibrotic metabolites such as reactive oxygen species (ROS). ROS are critical intermediates in both liver physiology and pathology. Recent research indicates that oxidative stress and the antioxidant system may also be critical for the development and persistence of fibrosis [8]. In addition to ROS, the transcription factor nuclear factor-kappa B (NF-*κ*B) is also essential for liver cell survival and liver homeostasis [9]. The regulation of cell death, inflammation, and wound healing by NF-*κ*B not only emphasizes the role of this transcription factor in the progression of liver diseases, but also highlights the mechanistic links between liver injury, inflammation, fibrosis, and hepatocellular carcinoma [10]. Several studies have indicated that NF-*κ*B inhibition is a potent mechanism for the induction of HSC apoptosis [11, 12]. Hence, when NF-*κ*B activation is prevented or inhibited, apoptosis of activated HSCs is enhanced.

**Figure 1:**
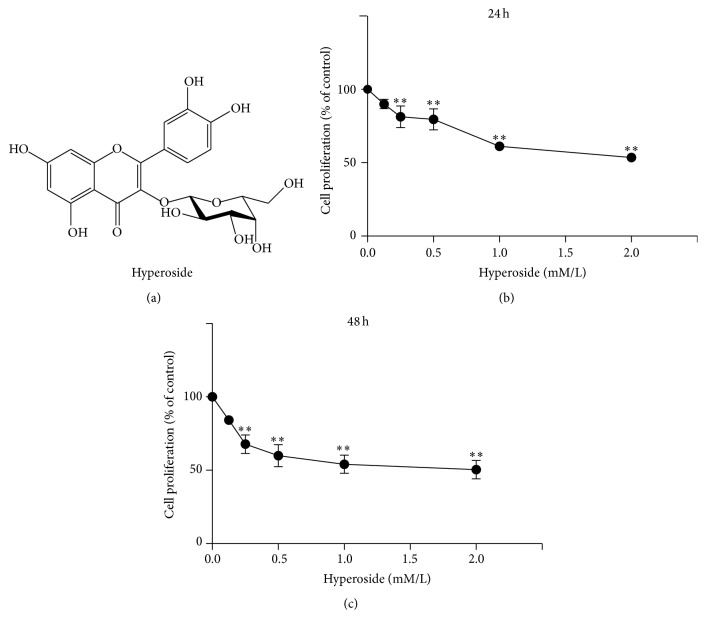
Structure of hyperoside and its inhibitory proliferation effect on LX-2 cells. (a) Chemical structure of hyperoside. (b) Effect of hyperoside on the growth of LX-2 cell lines for 24 h. (c) Effect of hyperoside on the growth of LX-2 cell lines for 48 h. Cell proliferation was analyzed using MTT assay. Cells were treated with different concentration of hyperoside (0, 0.125, 0.25, 0.5, 1.0, and 2.0 mM/L). Results represent the mean ± SEM from three independent experiments (^*∗∗*^means compared with the control group, *P* < 0.001).

Natural products have recently attracted much attention in drug research because they have frequently served as major sources of chemical diversity for novel biomedical agents and pharmaceutical discovery. Flavonoids are plant polyphenols found in vegetables, fruits, and plant-based beverages and are well known for their physiological antipyretic, analgesic, and anti-inflammatory activities [13]. Hyperoside (also called quercetin 3-O-b-d-galactoside; Figure 1(a)), a major pharmacologically active component from the genera* Hypericum* and* Crataegus *[14], has been demonstrated to possess numerous biological functions, including cardioprotective [15], antiredox [16], and anti-inflammatory activities [17]. Hyperoside also displays antiviral activity against hepatitis B in HepG2 cells transfected with hepatitis B viral genome, via the suppression of hepatitis B antigen secretion [18]. However, little research has been conducted on the potential roles and mechanisms of hyperoside in the treatment of liver fibrosis. In the present study, we utilized the human HSC line LX-2, which preserves key features of primary HSCs critical for liver fibrosis research [19], to investigate the molecular mechanisms of the proapoptotic effects of hyperoside.

## 2. Materials and Methods

### 2.1. Cell Culture

LX-2 cells were gifted by Dr. Zhigang Bai, Liver Research Center, Beijing Friendship Hospital, Beijing, China. Cells were cultured in RPMI-1640 medium (Thermo Fisher, Beijing, China) containing 10% fetal bovine serum at 37°C in a 5% CO_2_ incubator. Hyperoside (Biopurify, Chengdu, China) was dissolved in dimethyl sulfoxide (DMSO, Solarbio, Beijing, China) and added at the concentrations indicated.

### 2.2. Cell Viability Assay

3-(4,5-Dimethyl-2-thiazolyl)-2,5-diphenyl-2H-tetrazolium bromide (MTT, Sigma, M5655, USA) was used as an indicator of cell viability. Cells were grown in 96-well plates at a density of 5 × 10^3^ cells/well. After 24 h, the cells were washed with fresh medium, followed by treatment with hyperoside. After a 24 h or 48 h incubation period, the cells were washed, and 100 *μ*L of MTT (1 mg/mL) was added, followed by incubation for 4 h. Finally, DMSO (150 *μ*L) was added in order to solubilize the formazan salt formed, and the amount of formazan salt was determined by measuring the OD at 540 nm using a microplate reader (Synergy HT; BioTek Instruments).

### 2.3. Apoptosis Quantification

Detection of apoptosis was performed with an Annexin V-FITC/PI kit (BD Biosciences, New Jersey, USA) according to the manufacturer's instructions. Cells were seeded in 6-well plate (1 × 10^6^ cells/well) and treated with different concentrations (0.5 *μ*M, 1.0 *μ*M, or 2.0 *μ*M) of hyperoside at 37°C for 24 h or 48 h. Cells were harvested, washed twice with ice-cold PBS, and resuspended in 1 mL binding buffer. Resuspended cells (100 *μ*L) were transferred to a 1.5 mL EP tube and 5 *μ*L Annexin V-FITC plus 5 *μ*L PI was added. The tube was gently vortexed and incubated for 15 min at room temperature in the dark. Binding buffer (400 *μ*L) was then added and the cells were analyzed immediately by flow cytometry. Percentage of Annexin V-FITC^+^ cells was represented as apoptosis rate [20].

### 2.4. Western Blotting Analysis

Protein was extracted from LX-2 cells prepared with ice-cold lysis buffer (P1016, Solarbio Science and Technology, Beijing, China). Protein concentration in cell lysates was determined with a BCA protein kit (Beyotime, Shanghai, China). Samples containing equal amounts of protein (20 g) were mixed with loading buffer containing 5% 2-mercaptoethanol, heated for 5 min at 95°C, loaded onto a 10% SDS-PAGE gel, and then transferred to polyvinylidene difluoride (PVDF) membranes. After blocking with 5% milk and 0.1% Tween 20 in Tris-buffered saline (TBS), membranes were incubated overnight at 4°C with primary antibody. Antibodies against *α*-SMA (BM0002) and collagen I (BA0325) were purchased from Boster (Wuhan, China); *β*-tubulin (CW0098) was purchased from CWBIO (Beijing, China). Membranes were then incubated with the appropriate horseradish peroxide conjugated secondary antibody at room temperature. Protein bands were detected using ECL (WBKLS0100, Millipore, Billerica, MA, USA), according to the manufacturer's protocols. Experiments were repeated three times.

### 2.5. Intracellular Reactive Oxygen Species Assay

The production of intracellular reactive oxygen species was measured by DCFH oxidation. The DCF-DA reagent passively enters cells, where it is deacetylated by esterases into nonfluorescent DCFH. Inside the cell, DCFH reacts with ROS to form DCF, a fluorescent product. For this assay, 10 mM DCF-DA (S0033, Beyotime, Jiangsu, China) was dissolved in methanol and diluted 1000-fold in RPMI-1640 medium to give a final concentration of 10 *μ*M DCF-DA. Hyperoside treated LX-2-enriched cultures seeded (5 × 10^4^/well) in 96-well plates were then incubated with DCFH-DA for 20 minutes at 37°C. Immediately after incubation, DCF fluorescence was read at 485 nm excitation and 530 nm emission by flow cytometry.

### 2.6. NF-*κ*B DNA-Binding Activity

The Trans-AM NF-*κ*B p65 Transcription Factor Assay kit (Active Motif North America, Carlsbad, CA) was used to quantify the DNA-binding activity of NF-*κ*B in LX-2 cells, according to the instructions of the manufacturer. Briefly, nuclear extracts were prepared with NE-PER nuclear and cytoplasmic extraction reagents (Pierce Biotechnology Rockford, IL). Protein content in the two fractions was quantitated using a Bradford assay. Nuclear extracts were incubated in 96-well plates coated with immobilized oligonucleotide (5′-AGTTGAGGGGACTTTCCCAGGC-3′) containing a consensus (5′-GGGACTTTCC-3′) binding site for the p65 subunit of NF-*κ*B. NF-*κ*B binding to the target oligonucleotide was detected by incubation with primary antisera specific for the activated form of p65. The ELISA assay was developed with anti-IgG horseradish peroxidase conjugate and developing solution provided with the kit. Optical density (OD) was determined at 450 nm with a reference wavelength of 655 nm. Background binding was subtracted from the value obtained for binding to the consensus DNA sequence.

### 2.7. Quantitative RT-PCR

Total RNA was extracted from LX-2 cells using TRIzol Reagent (Invitrogen, Carlsbad, CA). A 10 *μ*g aliquot of each RNA sample was reverse transcribed into cDNA using oligo-dT random primers and reverse transcriptase. Quantitative real-time PCR was performed using SYBR Premix Ex Taq*™* II (Takara, Japan). The primers used are shown in Table 1. 18s was used as a housekeeping gene.

### 2.8. Statistical Analysis

Data are expressed as mean ± SEM, representing at least three independent experiments. Statistical analysis was conducted using SPSS software (SPSS version 14.0, SPSS Science, Chicago, IL, USA). One-way analysis of variance (ANOVA) was followed by least significant difference (LSD) test. A *P* value of less than 0.05 (*P* < 0.05) was considered to indicate statistical significance.

## 3. Results

### 3.1. Hyperoside Inhibits LX-2 Proliferation

The chemical structure of hyperoside is shown in Figure 1(a). The fibrogenic activities of HSCs are based on their activation and proliferation. To explore the potential antifibrotic effects of hyperoside, the viability of LX-2 cells following hyperoside treatment was determined by MTT assay. As shown in Figures 1(b) and 1(c), hyperoside treatment for both 24 h and 48 h significantly reduced LX-2 cell viability in a dose-dependent manner. The 50% inhibitory concentration (IC_50_) values for hyperoside were determined as 1.16 mM for 24 h treatment and 0.78 mM for 48 h treatment.

### 3.2. Hyperoside Promotes Apoptosis in LX-2 Cells

**Figure 2:**
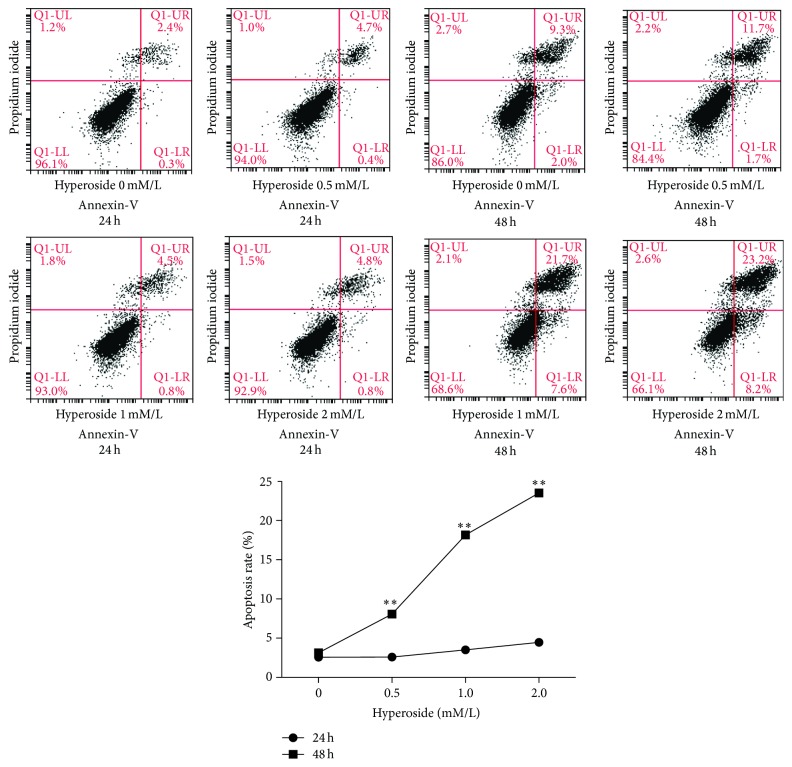
Hyperoside induced proapoptosis effect and its statistical representation of data. The apoptosis rate of Lx-2 cells was analyzed by flow cytometry (Annexin V-FITC/PI). Cells were treated with different dose of hyperoside (0, 0.5, 1.0, and 2.0 mM/L) for 24 h and 48 h, respectively. Results represent the mean ± SEM from three independent experiments (^*∗∗*^means compared with the control group, *P* < 0.001, *n* = 3).

To determine whether the decrease in cell viability we observed in hyperoside treated LX-2 cells was attributable to the induction of apoptosis, we examined the rate of LX-2 apoptosis using Annexin V-FITC/PI labeling.  Figure 2 showed that hyperoside treatment induced apoptosis in a concentration-dependent manner in LX-2 cells. The rate of apoptosis did not increase significantly in cells treated for only 24 h, while 48 h of hyperoside treatment significantly increased the rate of apoptosis in LX-2 cells. After 48 h of hyperoside treatment, the curvilinear response had an inflection point at the 0.5 mM dose, at which apoptosis rate was significantly increased. Because of these findings, we used hyperoside at a concentration of 1 mM in subsequent experiments involving the LX-2 cell line. These results indicate that growth inhibition of LX-2 cells by hyperoside is associated with apoptosis.

### 3.3. Hyperoside Downregulates Endogenous *α*-SMA and ECM Protein Levels in LX-2 Cells

**Figure 3:**
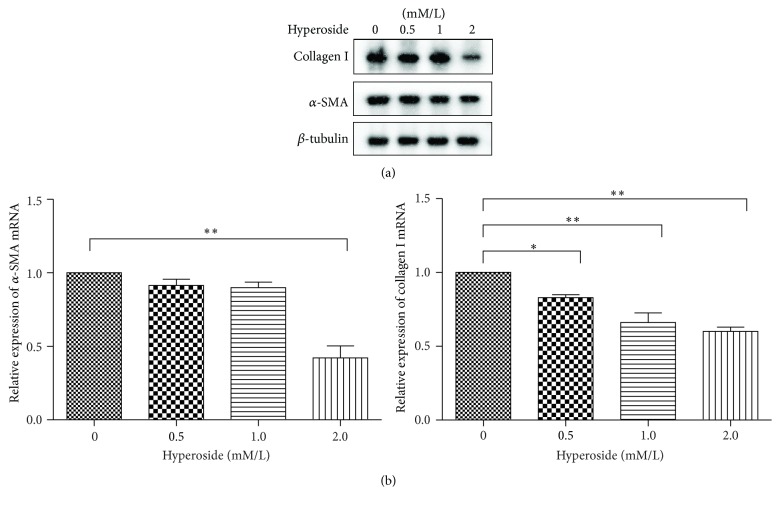
Hyperoside attenuated LX-2 activation. (a) Western blotting analysis of *α*-SMA and collagen I. The relative expression of proteins was calculated according to the reference band of *β*-tubulin. (b) mRNA levels were quantitated by real-time PCR. The expression was analyzed by 2^-ΔΔCT^ method. 18s mRNA was used as a housekeeping gene (^*∗*^means compared with the control group, *P* < 0.05; ^*∗∗*^means compared with the control group, *P* < 0.001, *n* = 3).

The major phenotypical transformation that follows HSC activation is the transdifferentiation into *α*-smooth muscle actin- (*α*-SMA-) positive myofibroblasts that have increased cell proliferation and produce large amounts of ECM proteins such as collagen I [5]. The effect of hyperoside on expression of the HSC activation markers *α*-SMA and type I collagen was evaluated by western blotting. LX-2 cells were exposed to hyperoside for 48 h, and total protein was isolated for subsequent analysis. As depicted in Figure 3(a), secretion of *α*-SMA was inhibited by hyperoside, but the effect was not statistically significant until treated with 2.0 *μ*M hyperoside. Treatment with 0.5 *μ*M hyperoside slightly suppressed the expression of collagen I levels, and the protein levels markedly decreased when the hyperoside is 1.0 *μ*M. As depicted in Figure 3(a), treatment with 0.5 *μ*M or 1.0 *μ*M hyperoside slightly decreased *α*-SMA and collagen I levels. Treatment with 2.0 *μ*M hyperoside markedly decreased *α*-SMA and collagen I protein levels.

Next, we quantified mRNA levels of *α*-SMA and collagen I using quantitative RT-PCR. As illustrated in Figure 3(b), *α*-SMA mRNA levels were significantly decreased in LX-2 cells treated with 2.0 *μ*M hyperoside. A significant decrease in collagen I gene expression was also observed. These results are consistent with the decreased protein levels shown in Figure 3(a).

### 3.4. Hyperoside Attenuates Intracellular ROS Production in LX-2 Cells

**Figure 4:**
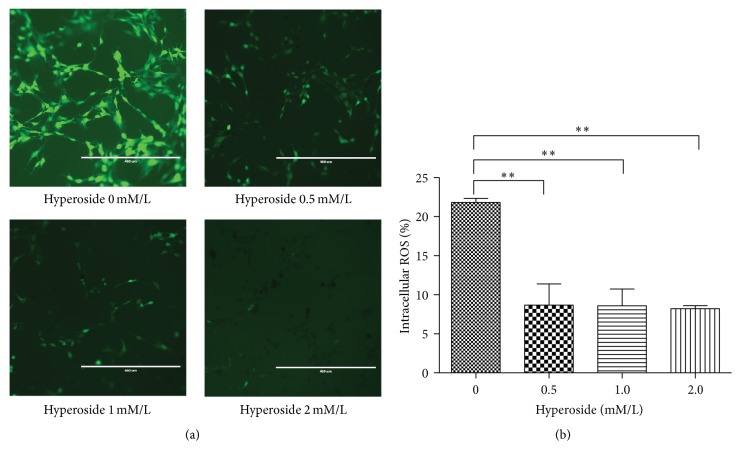
(a) LX-2 cells were treated in the absence or presence of hyperoside with different concentration for 24 h. The cells were then stained with DCF-DA and subjected to fluorescence microscopy. (b) Intracellular ROS was detected with flow cytometry.

Flavonoids are broadly recognized for their natural antioxidant properties [21], while ROS have been associated with fibrosis in several organs, especially in the liver [22, 23]. When we treated LX-2 cells with hyperoside at multiple concentrations and measured intracellular ROS levels by DCF-DA fluorescence, we found that hyperoside drastically reduced intracellular ROS production in activated liver fibrosis cells (Figure 4(a)). Quantitative evaluation of DCF fluorescence (Figure 4(b)) found that this inhibitory effect on intracellular ROS production was reduced by approximately 60% compared to control levels. We are the first to report that hyperoside attenuates intracellular ROS production in LX-2 cells.

### 3.5. Hyperoside Blocks NF-*κ*B Activation in LX-2 Cells

**Figure 5:**
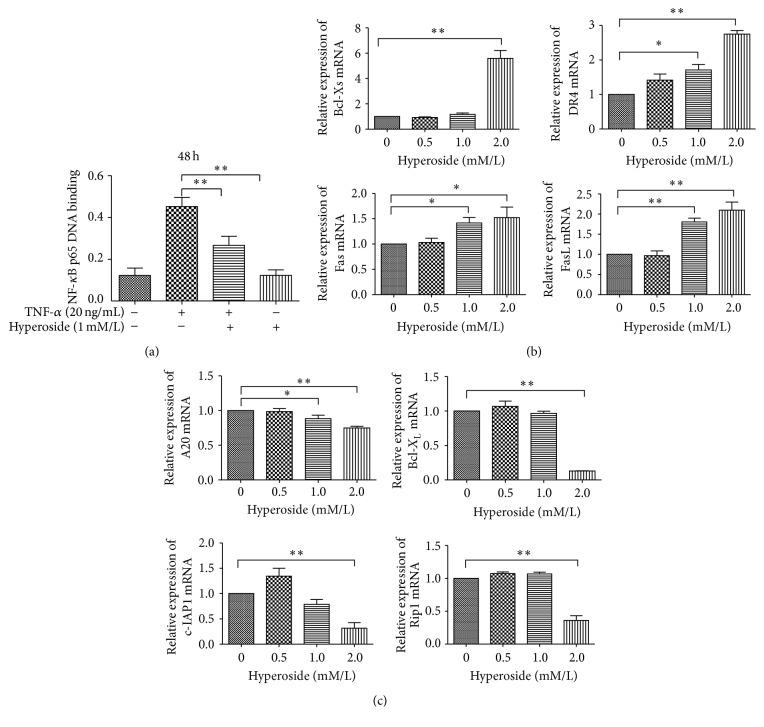
Hyperoside induces HSC apoptosis by blocking NF-*κ*B activation and mediating NF-*κ*B-dependent genes. (a) Hyperoside blocks NF-*κ*B activation in Lx-2 cells. The effect of hyperoside on NF-*κ*B DNA-binding activity in LX-2 cells was evaluated by ELISA. LX-2 cells were incubated for 48 hours in the absence or presence of TNF-*α* (20 ng/mL) and hyperoside (1 mM/L). Nuclear protein was extracted and subjected to ELISA for measurement of NF-*κ*B DNA-binding activity. (b) Relative expression of NF-*κ*B mediating proapoptotic target genes. (c) Relative expression of NF-*κ*B mediating antiapoptotic target genes (^*∗*^means compared with the control group, *P* < 0.05; ^*∗∗*^means compared with the control group, *P* < 0.001, *n* = 3).

To evaluate the NF-*κ*B-inhibiting effects of hyperoside, nuclear extracts were prepared from pretreated LX-2 cells and the subcellular localization of the NF-*κ*B RelA/p65 DNA-binding complex was examined. As shown in Figure 5(a), TNF-*α* was effective in inducing NF-*κ*B DNA-binding activity. Nuclear extracts from control LX-2 cells demonstrated high levels of NF-*κ*B DNA-binding activity, which was reduced by nearly 50% after hyperoside treatment when compared to the TNF-*α* induced group. This finding demonstrates that hyperoside can markedly attenuate TNF-*α* induced NF-*κ*B activation in LX-2 cells.

### 3.6. Hyperoside Induces HSC Apoptosis by Mediating NF-*κ*B-Dependent Genes

The NF-*κ*B transcription factor complex regulates genes governing a wide range of biological functions; these genes include regulators of apoptosis and cell proliferation such as* Fas*,* FasL*,* DR4*, and* Bcl-X*_*L*_ [24, 25], and NF-*κ*B mediates cell death by the upregulation or downregulation of these target genes. To gain further insight into the effects of hyperoside on the regulation of NF-*κ*B target genes in LX-2 cells, we assayed the expression of several proapoptotic genes (*Bcl-Xs*,* DR4*,* Fas*,* FasL*) and antiapoptotic genes (*A20*,* c-IAP1*,* Bcl-X*_*L*_,* RIP1*) by real-time PCR following hyperoside treatment. As shown in Figure 5(b), we detected a dose-dependent upregulation of proapoptotic genes after 48 h of hyperoside treatment, with the largest increases in mRNA levels observed in* DR4* and* FasL*. Conversely, the expression of antiapoptotic genes* Bcl-X*_*L*_,* c-IAP1*, and* Rip1 *decreased with increasing doses of hyperoside.

## 4. Discussion

Recently, naturally occurring plant compounds have become attractive subjects of research into novel therapeutic strategies for liver fibrosis. In particular, dietary flavonoids such as morin [26], hesperidin [27], and silymarin [28] have been reported to attenuate the process of fibrosis. Hyperoside has been shown to have pleiotropic pharmacological effects, which include antithrombotic activities [29], protective effects on cardiomyocytes [30], and anti-inflammatory activities [31]. A recent report suggested that hyperoside exhibits hepatoprotective effects against CCl_4_-induced liver injury and suppresses inflammation, but the mechanisms by which it protects against liver cirrhosis still need to be elucidated [32]. In the present study, we demonstrated that hyperoside induced culture-activated HSCs to undergo apoptosis due to downregulation of intercellular ROS and thereby inhibition of NF-*κ*B.

NF-*κ*B signaling plays an active role in a number of chronic liver diseases [10, 33]. There is evidence of NF-*κ*B activity in activated HSCs [6, 34] and the inhibition of HSC apoptosis promotes liver fibrosis [7, 11, 35]. Inhibition of NF-*κ*B signaling is usually connected with the induction of apoptosis in activated HSCs and the resolution of experimentally induced liver fibrosis [36]. For example, gliotoxin [7] and resveratrol [37] can induce HSC apoptosis both* in vitro* and* in vivo *through the inhibition of NF-*κ*B signaling and altered regulation of NF-*κ*B-dependent gene transcription [38]. Consistent with these reports, we observed that when hyperoside was administered to LX-2 cells for 48 h, late apoptotic and necrotic cells (Figure 2) were increased significantly. In the early apoptosis stage, cells trigger ultimate loss of the mitochondrial membrane potential and translocation of phosphatidylserine. Early apoptotic cells can be rescued from the apoptotic program if the apoptotic stimulus is removed [39]. In our study, for early and median apoptotic cells, there is slighter change in 24 h than in 48 h perhaps for LX-2 cells 24 h hyperosdie treatment is not as effective as 48 h, which is consistent with the result of cell viability assay that for 48 h IC_50_ is lower than that in 24 h. As a consequence of NF-*κ*B signaling inhibition, active markers for HSCs *α*-SMA and type I collagen were reduced by hyperoside in LX-2 cells.

Theoretically, ROS represent a serious hazard for the cell, as not only can they oxidize macromolecules—thus damaging proteins, lipids, and DNA [40]—but they are also key secondary messengers in numerous signaling pathways including proliferation, metabolism, and apoptosis [41]. In the liver, ROS contribute to hepatic fibrosis triggered by numerous liver injuries, including alcohol abuse, HCV infection, iron overload, and chronic cholestasis [42, 43]. Importantly, several studies have found that ROS influence intracellular NF-*κ*B signaling [44] and stimulate collagen gene induction in HSCs, contributing to the pathogenesis of liver fibrosis [45]. Interestingly, studies have revealed the existence of a reciprocal, negative feedback loop between NF-*κ*B and ROS [41]. Hyperoside is a quercetin-3-O-galactoside compound with a catechol group in the B-ring. Based on established structure-antioxidant activity relationships [46], we hypothesized that hyperoside would exert the suppressive capacity of intercellular ROS in HSCs. As expected, our experimental results indicate that hyperoside attenuates the generation of intracellular ROS and decreases the activation of NF-*κ*B.

Collectively, our results suggest that the antifibrotic effects of hyperoside on cultured LX-2 cells are mediated by the inhibition of canonical NF-*κ*B signaling and the induction of apoptosis in activated HSCs. Hyperoside can be regarded as a potential candidate in the search for pharmacological agents to combat liver fibrosis, although the precise mechanisms involved remain to be discovered, and extensive preclinical experiments are still required. Plants possessing the hyperoside compound are abundant in China [47], and many of these are considered to be atoxic and edible according to traditional Chinese medicine. This study adds support to the notion that the development of novel medicines and health-promoting foods based on hyperoside is a viable strategy, especially in China, and represents a valuable research goal because liver cirrhosis arising from viral hepatitis remains a serious global health issue.

## Figures and Tables

**Table 1:** Primers of quantitative real-time PCR.

Primers	Sequence		Annealing temperature (°C)
	Forward (from 5′ to 3′)	Reverse (from 5′ to 3′)	
18s	CAGCCACCCGAGATTGAGCA	TAGTAGCGACGGGCGGTGTG	60
*α*-SMA	AGGTAACGAGTCAGAGCTTTGGC	CTCTCTGTCCACCTTCCAGCAG	60
Collagen I	GAGCGGAGAGTACTGGATCG	GTTCGGGCTGATGTACCAGT	60
A20	GTCCGGAAGCTTGTGGCGCT	CCAAGTCTGTGTCCTGAACGCCC	55
Bcl-Xs	GCAGTAAAGCAAGCGCTGAG	GTTCCACAAAAGTATCCTGTTCAAAG	60
DR4	CTGAGCAACGCAGACGCGCTGTCCAC	ACAGCATCAGAGTCTCAGTGGGGTCAGC	60
Fas	ATTCTGCCATAAGCCCTGTC	TTGGTGTTGCTGGTGAGTGT	55
FasL	GTTCTGGTTGCCTTGGTAGG	TGTGCATCTGGCTGGTAGAC	55
c-IAP1	TTGTCAACTTCAGATACCACTGGAG	CAAGGCAGATTTAACCACAGGTG	60
Bcl-X_L_	AGTTCCCTTGGCCTCAGAAT	AGGGTTGCACCAATCAGGTA	55
Rip1	GTCAAATTCAGCCACAGAACAGCC	CCCTTTAGCCTTCCCTCATCACC	55
